# Electrospun polyacrylonitrile-polyphenyl/magnetite nanofiber electrode for enhanced capacitance of supercapacitor

**DOI:** 10.1038/s41598-025-97089-x

**Published:** 2025-04-28

**Authors:** El-Refaie Kenawy, Youssef I. Moharram, Fatma S. Abouharga, Mona Elfiky

**Affiliations:** 1 Polymer Research Group, Department of Chemistry, Faculty of Science, Tanta University, Tanta, 31527 Egypt; 2 Analytical and Electrochemistry Research UNIT, Department of Chemistry, Faculty of Science, Tanta University, Tanta, Egypt

**Keywords:** Electrospinning process, High cycle life, Electrode materials, Cycling stability, Sustainable development., Energy science and technology, Energy storage, Supercapacitors

## Abstract

Supercapacitors are widely valued for their high cycle life, power density, and broad applications. However, the development of improved devicess hindered by the challenges related to electrode materials. Effective electrodes need high specific capacitance and low electrical resistance to enhance energy storage, while also being simple to prepare, cost-effective, and environmentally friendly to support sustainable development. This study utilized an affordable and straightforward electrospinning process to produce polyacrylonitrile (PAN) nanofibers, polyacrylonitrile-polyphenyl (PAN-PPh) nanofibers, and polyacrylonitrile-polyphenyl/magnetic iron oxide (PAN-PPh/Fe_3_O_4_) composite nanofibers for supercapacitor electrodes. Among these, the PAN-PPh/Fe_3_O_4_ electrode exhibited superior performance, with a specific capacity of 0.258 Ah g^− 1^, and specific capacitance of 442.4 F g^− 1^ and excellent cycling stability, retaining approximately 78.49% of its capacitance after 3000 cycles. These results highlight the potential of PAN-PPh/Fe_3_O_4_ composites as sustainable materials for supercapacitor electrodes.

**Supplementary Information:**

The online version contains supplementary material available at 10.1038/s41598-025-97089-x.

## Introduction

Energy profoundly plays a crucial role in our social and economic development and quality of life. Consequently, there has been extensive research has focused on electrochemical energy storage technologies, such as supercapacitors and rechargeable batteries. Supercapacitors, also known as ultracapacitors, are distinguished by their ability to store and transfer energy rapidly, delivering high currents in short bursts^1^. They are capable of charge and discharge quickly at high rates, have a longer lifespan, and are resistant to temperature variations, making them reliable and efficient^2^. Due to these advantages, supercapacitors are primarily used in heavy equipment and other high-power applications. They are composed of two electrodes, an electrolyte, and a separator, with the electrodes significantly influencing their performance characteristics. Among energy storage technologies, supercapacitors hold significant potential for future development and application^3^. Supercapacitors are categorized into two types based on their energy storage methods: electrochemical double-layer capacitors (EDLCs) and pseudocapacitors^4–7^. EDLCs store energy through electrostatic interactions between ions on a large specific surface area of active electrode materials and electrolytes. This mechanism allows EDLCs to undergo extremely fast charge-discharge cycles, completing over 100,000 cycles in seconds. The most promising materials for EDLC electrodes are carbon-based, such as graphene and its derivatives, carbon nanotubes (CNTs), activated carbon, and others^7^. An EDLC features a structure approximately 1 nm thick, consisting of a compact Helmholtz layer, a diffusive layer in the electrolyte, and a space charge layer in the electrode. The performance of an EDLC is largely determined by the electrochemical activity and kinetic properties of the electrode, necessitating electrode materials with high specific surface areas, significant porosity, and optimal pore distribution for high performance. In contrast, pseudocapacitors store energy through rapid and reversible Faradaic redox reactions occurring both on the surface and within the bulk of the electrode materials. Pseudocapacitors typically offer higher capacitance per gram than EDLCs, but their kinetics are slower because the energy storage process occurs in both the bulk and surface of the electrode materials. While EDLCs confine the charge/discharge process to the electrode surface, pseudocapacitors involve a more extensive reaction mechanism^8^. Supercapacitor performance can be enhanced by combining the advantages of various materials, such as conducting polymers and metal oxides. Pseudocapacitors often utilize conducting polymers (CPs) and metal oxides, especially transition metal oxides, to achieve superior performance.

Moreover, conductive polymers (CPs) have emerged as a promising class of electrode materials due to their high capacitance, superior flexibility, large surface area, excellent conductivity,  lightweight nature, and low cost. These materials store chemical energy through redox reactions, making them ideal candidates for stretchable supercapacitor electrodes^9^. Through π-electron delocalization along their polymer backbones, various conjugated polymers with alternating single and double bonds can now be synthesized to exhibit novel electrical properties. Conductive polymers such as polyaniline (PANI), polypyrrole (PPy), polythiophene (PTh), poly(3,4-ethylenedioxythiophene) (PEDOT), and polyacrylonitrile (PAN) have garnered significant interest recently due to their unique structures and properties^10^. Recently, supercapacitors have been developed using conductive polymers and Fe_3_O_4_^11,17^. Vinay S. Patil and colleagues created a novel supercapacitor using a magnetite (Fe_3_O_4_)-NH-polyaniline (PANI) composite. They successfully synthesized and tested a PANI-grafted Fe_3_O_4_ composite material for supercapacitor applications. The grafting of PANI chains onto magnetite helped overcome the limitations of composite materials formed by simply mixing metal oxide with conducting polymer. Adding reduced graphene oxide (rGO) further enhanced the supercapacitive performance of this composite. The Fe_3_O_4_-NH-PANI nanocomposite exhibited a specific capacitance of 191 F g^− 1^, a specific energy of 9.3 Wh kg^− 1^, and a specific power of 458.3 W kg^− 1^ at a scan rate of 10 mV.s^− 1^. With the inclusion of rGO, the rGO-Fe_3_O_4_-NH-PANI composite achieved a specific capacitance of 336.4 F g^− 1^, a specific energy of 11.81 Wh kg^− 1^, and a specific power of 1258.3 W kg^− 1^ at the same scan rate. Additionally, the polymer-grafted material-rGO composite demonstrated excellent cycle stability. Polyacrylonitrile (PAN) has been widely employed for producing nanofibers due to its high stability, predictable mechanical strength, low cost, and excellent spinnability^18^.

Electrospinning is a well-established method for producing continuous nanofibers with uniform diameters ranging from sub-micrometer to a few nanometers, and large surface areas^18^. In recent years, electrospinning has gained considerable attention as a low-cost, scalable, and straightforward technique for nanofiber production. By modifying the spinneret design, collection method, spinning conditions, and precursor materials, as well as by controlling the nanofiber structure, the performance of electrospun nanofiber electrodes can be significantly enhanced. Techniques such as using hollow or highly porous nanofibers have been explored to improve electrochemical properties^19^. Polyacrylonitrile (PAN) is frequently used as a precursor polymer for electrospinning nanofibers due to its strong spinnability and ease of processing^20^. However, pure PAN-based fibers  exhibit a low specific surface area, resulting in poor capacitance^21^. There has been Limited research has been conducted on combining PAN with polyphenyl Shuai Ru et al.^22^ successfully created PAN-based composites by producing and annealing N-doped carbon-coated Fe_3_O_4_ composites (Fe_3_O_4_@NC) using a polyacrylonitrile phase inversion process. The Fe_3_O_4_@NC-600 composite demonstrated superior electrochemical characteristics compared to pure Fe3O4 nanoparticles, thanks to the synergistic effect between the carbon and Fe_3_O_4_ nanoparticles. When used as an anode material in lithium-ion batteries (LIBs), the Fe_3_O_4_@NC composite exhibited a high specific capacity of 981 mAh g^− 1^ at 0.2 A.g^− 1^ after 500 cycles, excellent rate capability, and improved long-term high-rate cycling performance (632 mAh g^− 1^ at 1 A.g^− 1^ after 900 cycles). These results highlight the potential of the Fe_3_O_4_@NC-600 electrode as a high-performance anode for LIBs. Additionally, metal oxides are widely used in supercapacitors due to their high energy and power densities, as well as their high specific capacitance and conductivity^23^. The rapid and reversible redox reactions occurring on the electrode surface enable the charge storage process in pseudocapacitors. Transition metal oxides (TMOs), such as RuO_2_, Co_2_O_3_, Fe_2_O_3_, MnO_2_, V_2_O_5_, NiO, SnO_2_, IrO_2_, and MoO, as well as conducting polymers like polyaniline, polythiophene, and polypyrrole, are known for their redox behavior. Despite their increased energy density, TMOs often suffer from cyclic instability due to phase and distortion variations caused by ongoing reactions. The electrochemical performance of TMOs is influenced by factors such as crystallinity, pore size, active surface area of the electrode materials, and the size of the active redox species. Therefore, developing high energy and power density transition metal oxide nanostructures is crucial^24^.

Magnetite nanoparticles (Fe_3_O_4_) have gained significant attention for their large adsorption surface area, high chemical stability, exceptional magnetic properties, ease of separation, and nontoxicity,  making them useful in wastewater treatment, energy storage, and antibacterial activities. Fe_3_O_4_ is particularly attractive due to its low cost and environmentally friendly properties. However, low conductivity limits its rate capability when used as a supercapacitor electrodewhen used as a supercapacitor electrode, hindering rapid electron transport required for high-rate applications^25^. Fe_3_O_4_ exhibits unique valence states and undergoes reversible redox reactions. Brousse developed Fe_3_O_4_ powders with large surface areas, reporting a specific capacitance of 75 ± 8 F g^− 1^, while nano-sized cellular Fe_3_O_4_ thin sheets demonstrated a specific capacitance of up to 105 F g^− 1^^26^. Extensive research has been conducted on Fe_2_O_3_ and Fe_3_O_4_ based nanostructured materials for supercapacitors. However, bare Fe_2_O_3_ has limited applications due to its low electrical conductivity (10–14 S.cm^− 1^) and slow ionic diffusion rate, resulting in a specific capacitance much lower than the theoretically anticipated value^27^. Several strategies, such as creating Fe_2_O_3_ composite electrodes using conductive polymers, have been employed to overcome these challenges and enhance the performance of metal oxide-based supercapacitors.

In this work, we explored the unique composite nanofiber formed by electrospinning a combination of polyacrylonitrile (PAN), polyphenyl (PPh), and Fe_3_O_4_ nanoparticles to assess their performance as supercapacitors. PAN and PPh nanofibers were simultaneously electrospun to incorporate Fe_3_O_4_ nanoparticles. The resulting composite nanofibers were characterized using various techniques, including scanning electron microscopy (SEM), scanning transmission electron microscopy (STEM), Fourier transform infrared spectroscopy (FTIR), thermogravimetric analysis (TGA), X-ray diffraction (XRD), and energy-dispersive X-ray spectroscopy (EDX). The electrochemical performance of the composite nanofiber electrodes was evaluated using electrochemical impedance spectroscopy (EIS), cyclic voltammetry (CV), and charge-discharge experiments to determine their capacitance characteristics.

## Experimental

### Materials

Pure reagent-grade chemicals were obtained and used as received: ferric chloride (FeCl_3_, purum, anhydrous, ≥ 97.0%), iron(II) sulfate heptahydrate (FeSO_4_.7H_2_O, ACS reagent, ≥ 99.0%), polyacrylonitrile (PAN, MW = 150,000), N, N-dimethylformamide (DMF, SAJ first grade, ≥ 99.0%), benzene (C_6_H_6_, ACS reagent, ≥ 99.0%), aluminum chloride (AlCl_3_, purum, anhydrous, ≥ 98.0%), cupric chloride (CuCl_2_, ACS reagent, ≥ 99.0%), hydrochloric acid (HCl, ACS reagent, 37%), sodium hydroxide (NaOH, ultra dry, powder or crystals, 99.99%), perfluorinated resin solution ((Nafion) perfluorinated resin solution, 5 wt% in mixture of lower aliphatic alcohols and water, contains 45% water), isopropyl alcohol, (ISA ≥ 99.7%, fragrance grade), and potassium hydroxide (KOH, ACS reagent, ≥ 98.0%) were supplied by “Sigma-Aldrich”.

### Instruments

Fourier transform infrared (FT-IR) spectra of all the manufactured nanofiber samples were obtained using a Bruker TENSOR 27-series FTIR (Germany) in the range of 400–4000 cm^−1^. Thermal stability, maximum degradation temperature, and mass variation with increasing temperature were measured using a Perkin Elmer 4000 thermal analyzer with a heating rate of 10.0 °C/min in the range of 50–800 °C. Moreover, the morphological and chemical composition of the fabricated samples were analyzed using Scanning Electron Microscopy (SEM) and Energy Dispersive X-ray Spectroscopy (EDX) with a JEOL JSM 6510LV instrument, as well as Scanning Transmission Electron Microscopy (STEM) employing a Quattro S system from Thermo Fisher Scientific (USA). Cyclic voltammetry (CV), electrochemical impedance spectroscopy (EIS), and charge-discharge measurements were conducted for all samples using a computer-controlled potentiostat/galvanostat model CS3104 (China) in the microanalysis unit at the Faculty of Science, Tanta University. Additionally, XRD patterns of all produced materials were examined at Tanta University’s central laboratory using XRD equipment (300 Unisantis, Germany) with Cu-Kα radiation (λ ≈ 1.5406 Å, scanning rate of 0.05°/sec at 45 kV and 0.8 mA). The morphology and chemical composition of the produced samples were analyzed using SEM and EDX equipment (JEOL Japan, JSM 6510LV) and STEM (Quattro S, ThermoFisher, USA).

### Synthesis of magnetite nanoparticles

Magnetite nanoparticles (MNPs) were synthesized by co-precipitating ferric and ferrous ions in an alkaline solution. The procedure was as follows: 6.5 g of FeCl₃ and 5.56 g of FeSO₄·7 H₂O, representing a 1:2 molar ratio of Fe²⁺ to Fe³⁺, were dissolved in 50.0 ml of 0.5 M HCl solution. This solution was then mixed with 500.0 ml of 1.5 M NaOH added dropwise under vigorous stirring (600 rpm) and heated to 80 °C. The reaction environment had a pH value of 14. A dark precipitate of Fe₃O₄ formed immediately, indicating the formation of magnetite nanoparticles. The reaction proceeded rapidly upon the addition of iron salts, with the magnetite nanoparticles becoming visible instantly. The paramagnetic properties were confirmed by placing a magnet next to the black precipitate, which facilitated the separation of the precipitate in the magnetic field. The supernatant liquid was decanted multiple times with distilled water to neutralize the pH of the solution. The precipitate was then dried at 50 °C for 4 hours and overnight at room temperature, resulting in MNPs in powder form. The particle size was measured using SEM analysis^28^.

### Synthesis of p-polyphenyl

p-Polyphenyl was synthesized using a benzene-aluminum chloride-cupric chloride mixture, with strict precautions to avoid contamination. The reaction was carried out in a three-necked flask equipped with a paddle stirrer and under a N₂ atmosphere. The procedure was as follows: a mixture of benzene, AlCl₃, and CuCl₂ in a molar ratio of 1:0.5:0.5 was prepared and injected into the flask. The temperature increased to 37 °C, and the reaction mixture was continuously stirred in the presence of an acidic gas for 30 min. After the reaction, deionized water (DW) was added to the mixture, which was then filtered. The product was subsequently treated with an 18.0% diluted HCl solution, heated with concentrated HCl, and rinsed with DW until the washings were colorless. The polymer was further purified by boiling twice with a 2.0 M NaOH solution, followed by thorough rinsing with DW until the washings were colorless and free from chloride ions. Finally, the polymer was dried at 120 °C for 5 hours, yielding a finely divided, light brown powder^29,30^.

### Preparation of polyacrylonitrile nanofibers

A 10 wt% polyacrylonitrile (PAN) solution was prepared by dissolving 1.0 g of PAN in 10 ml of DMF. The mixture was stirred steadily for 2 hours at 80 °C to disrupt the strong intra- and interchain bonding in the PAN polymer. The resulting polymer solution, with a viscosity of 8703 cP (8.703 Pa.s), was then loaded into a 10 ml plastic syringe fitted with a 0.4 mm diameter needle. For the electrospinning process, a positive electrode (anode) was connected to the syringe tip, while a negative electrode (cathode) was attached to a metallic collector wrapped in aluminum foil. The distance between the syringe tip and the collector was set to 15 cm, and the flow rate was maintained at 10 ml/min. An applied potential of 14 kV was used to facilitate the electrospinning of the PAN nanofibers^31^.

### Preparation of polyacrylonitrile and polyphenyl nanofibers

To prepare the nanofibers, a solution containing 2.08 wt% polyphenyl (PPh) and 8.33 wt % polyacrylonitrile (PAN) was made. First, 25.0 g of PPh was sonicated for 1 hours in 12 ml of N, N-dimethylformamide (DMF). Following this, 1 g of PAN was added to the sonicated solution, and the mixture was stirred continuously for 2 h at 80 °C. The resulting composite solution, with a viscosity of 2838 cP (2.838 Pa.s), was then loaded into a 10-ml plastic syringe fitted with a 0.4 mm diameter needle. For the electrospinning process, a positive electrode (anode) was connected to the syringe tip, and a negative electrode (cathode) was attached to a metallic collector wrapped in aluminum foil. The syringe tip-to-collector distance was set to 15 cm, with a flow rate of 10 ml/min and an applied voltage of 14 kV to facilitate the formation of the nanofibers^32^.

### Preparation of nanofiber composite by electrospinning technique

To prepare the nanofiber composite, a solution containing 4.17 wt% Fe_3_O_4_, 2.08 wt% polyphenyl (PPh.), and 8.33 wt% polyacrylonitrile (PAN)weres made. First, 0.5 g of Fe_3_O_4_ nanoparticles and 0.25 g of PPh. were sonicated in 12 ml of N, N-dimethylformamide (DMF) for 1 hour to ensure proper dispersion. Subsequently, 1 g of PAN was added to the sonicated solution and stirred continuously for 2 hours at 80 °C. The resulting composite solution, with a viscosity of 1659 cP (1.659 Pa.s), was then loaded into a 10 ml plastic syringe fitted with a 0.4 mm diameter needle. For the electrospinning process, a high voltage of 14 kV was applied to the solution, and the fibers were collected on a metallic collector. The distance between the syringe tip and the collector was set to 15 cm, with a flow rate of 10 ml/min to facilitate the formation of the nanofibers^33^.

### Electrochemical measurements

A glassy carbon electrode (GCE) with a diameter of 3.0 mm was polished using 0.05 μm alumina powder to achieve a mirror-like finish and was subsequently cleaned thoroughly. Following this, 0.1 mg of PAN-PPh./Fe_3_O_4_ nanofiber was deposited onto the GCE surface. A 5 µl mixture of 1.0 ml nafion and 1.0 ml isopropyl alcohol was then applied to the sensor surface, and the PAN-PPh./Fe_3_O_4_ nanofibers were allowed to dry at 60 °C for 2 hours. A similar procedure was employed to fabricate [PAN] and [PAN-PPh.] nanofiber GCEs. Electrochemical measurements were conducted in a 10.0 ml 1.0 M KOH solution using a three-electrode configuration with Hg/Hg_2_Cl_2_ and platinum electrodes (Pt) as the reference and counter electrodes, respectively. CV was performed within a potential range of -1.50 V to 0.50 V at a scan rate of 50 mV/s, while electrochemical impedance spectroscopy (EIS) was conducted over a frequency range of 0.1 to 106 Hz. Specific capacitance (Cs) values for [PAN], [PAN-PPh.], and [PAN-PPh./Fe_3_O_4_] nanofiber GCEs were calculated using Eqs. (1) and (2) as mentioned in^34^:

1 $$Specific~capacity~\left( {Ah/g} \right)~\left( {CV~analysis} \right)=\frac{{\smallint i\left( V \right)dV\left( {AV} \right)~}}{{m\left( g \right)~v\left( {V.s - 1} \right)*~3600}}$$

2 $$Specific~capacity~\left( {Ah/g} \right)~\left( {GCD~analysis} \right)=\frac{{\smallint i\left( A \right)dt\left( s \right)~}}{{m\left( g \right)*~3600}}~$$

In the equations provided, *v*/mV/s denotes the scan rate, while ΔV/V, m/ mg, i/A, and t/s represent the applied potential range, the mass of the sample on the surface of sensor, the current value during charge-discharge, and the duration of charge-discharge, respectively.

## Results and discussion

### Characterization of prepared nanofibers

#### FT-IR and XRD analysis of nanofibers

**Fig. 1:**
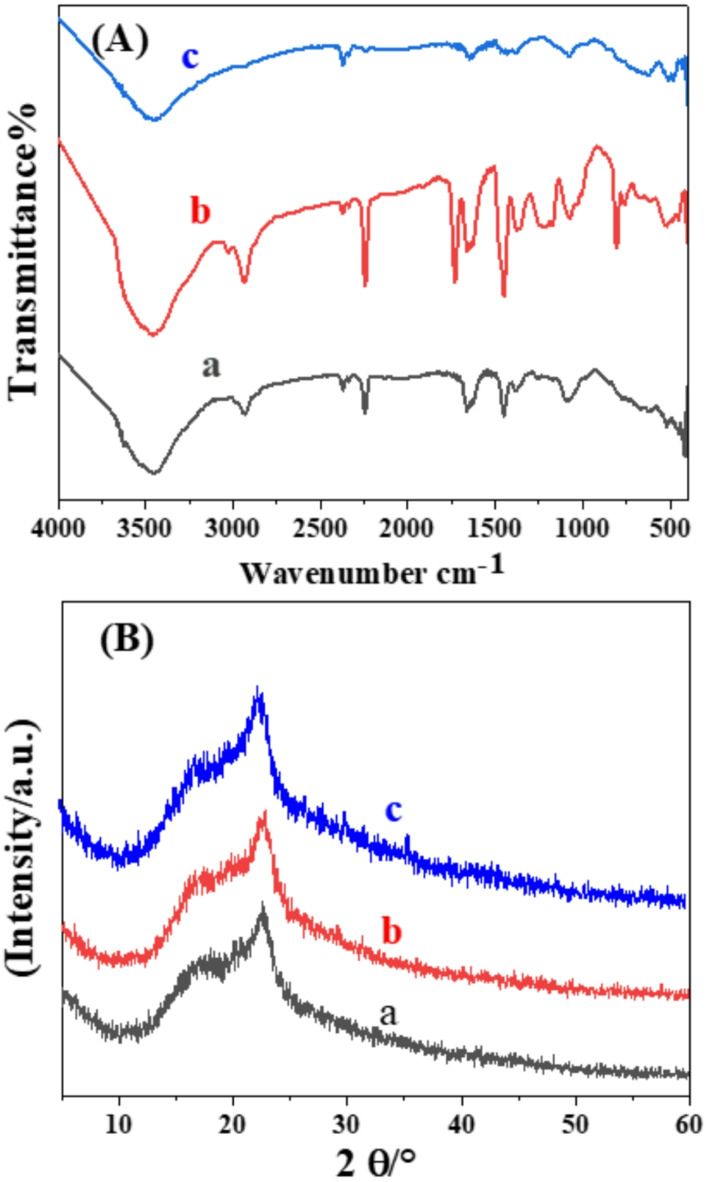
(**A**) FT-IR spectra and (**B**) XRD patterns of (a) PAN, (b) PAN-PPh. and (c) PAN-PPh./Fe_3_O_4_ nanofibers.

FTIR spectra of PAN, PAN-PPh., and PAN-PPh./Fe_3_O_4_ nanofibers were obtained to provide detailed information about the electrospun materials^35^ as illustrated in Fig. 1A. The spectra display prominent broad bands in the 3640–2500 cm^−1^ range and distinct peaks at 2933.2 cm^−1^ and 2870 cm^−1^, attributed to stretching *v*_OH_, and asymmetric and symmetric *v*_C−H_ in CH, CH_2_, and CH_3_ groups across all nanofibers^36^, respectively. The FTIR spectrum of PAN nanofiber reveals absorption bands at 2245, 1450.21, 1378.85, 1240, and 1088.62 cm^−1^, which are associated with the stretching of *v*_C≡N_, *v*_CH3_, and *v*_CH2_ groups, as well as symmetric stretching of *v*_CH3_ in stretching *v*_C–N_, and bending *v*_C–N_, as shown in Fig. 1A_a_. In addition, the spectrum of PAN-PPh. Figure 1A_b_ shows absorption bands at 1648, 1590, 1488, and 1450 cm^−1^, attributed to aromatic *v*_C−C_, with a strong band at 1220 cm^−1^ corresponding to the stretching of Ph-O-Ph in aromatic ether chains^36^. The FTIR spectrum of PAN-PPh./Fe_3_O_4_ nanofiber (Fig. 1A_c_) shows minor shifts in these bands, indicative of interactions among the nanofiber components. Additionally, the FTIR spectra of Fe_3_O_4_, as reported in several studies^37^, show bands at 427, 479, 507, and 627 cm^−1^, corresponding to Fe-O vibrations^38^.

The crystallinity of PAN, PAN-PPh., and PAN-PPh./Fe3O4 nanofibers was further investigated using X-ray diffraction (XRD) patterns, as shown in Fig. 1B. The XRD pattern of PPh. exhibits well-defined diffraction peaks at 2θ ≈ 19.9°, 22.78°, 28.0°, and 43.0° (Figure S1), while Fe_3_O_4_ shows distinct peaks at 2θ ≈ 32.0°, 35.8°, and 45.9° (Figure S_2_). The XRD pattern of PAN nanofibers^39^ reveals broad peaks at 2θ ≈ 16.9° and 22.5° (Fig. 1B). Notably, the PAN-PPh./Fe_3_O_4_ nanofiber pattern (Fig. 1B) presents a new sharp peak at 2θ ≈ 35.7°, which can be associated with Fe_3_O_4_ nanoparticles.

#### Investigation of morphological structure

**Fig. 2:**
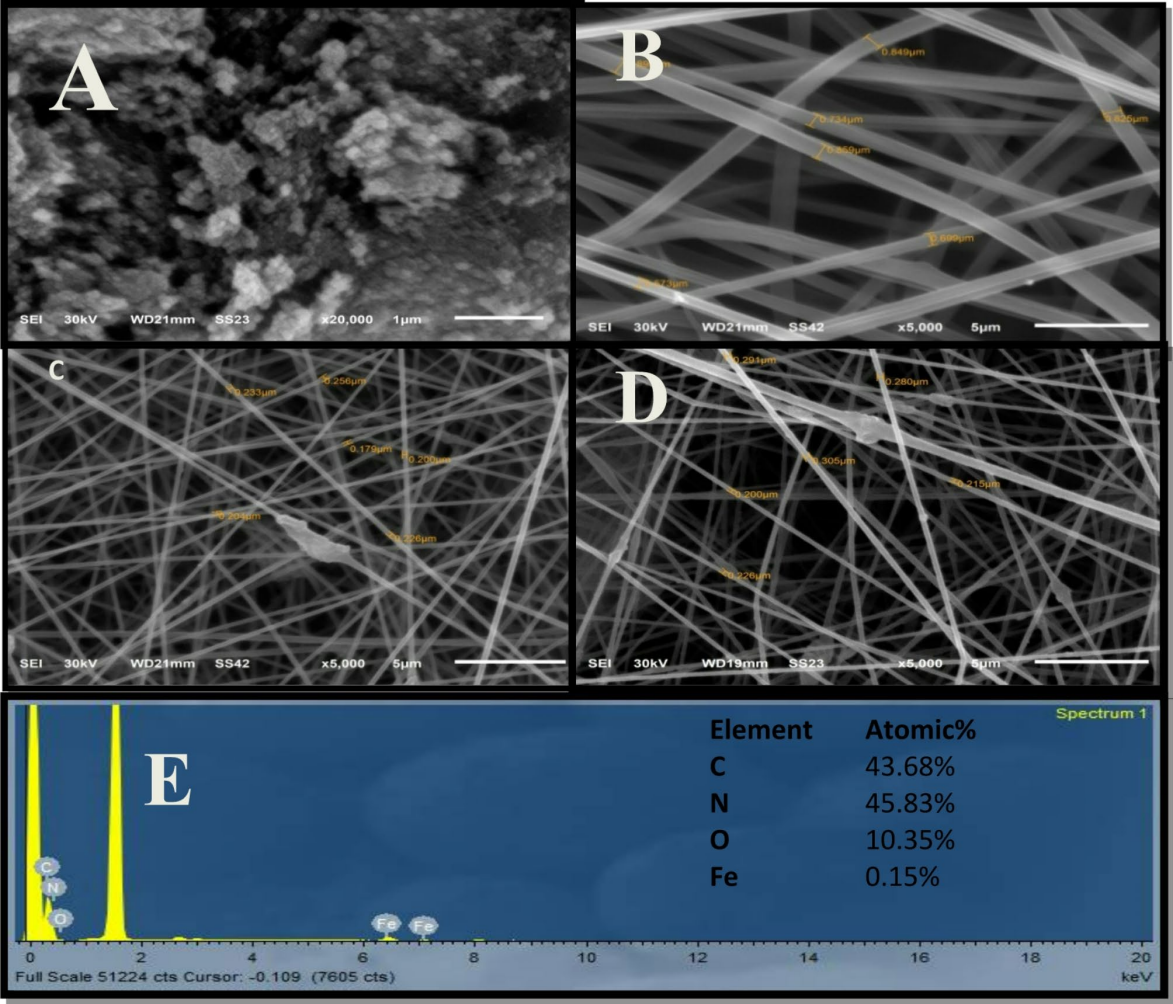
SEM images of (**A**) Fe_3_O_4_ NPs, (**B**) PAN, (**C**) PAN-PPh., and (**D**) PAN-PPh./Fe_3_O_4_ nanofiber. (**E**) EDX spectrum of PAN-PPh./Fe_3_O_4_ nanofiber.

Figure 2A presents a scanning electron microscopy (SEM) image of Fe_3_O_4_ nanoparticles, which are clearly spherical with an average diameter of 23 nm measured by using (ImageJ 1.42q software). As illustrated in Fig. 2B, C, D, the average diameters of the prepared nanofibers are measured as 921.02 nm, 217.99 nm, and 211.41 nm for PAN nanofibers, PAN-PPh., and PAN-PPh./Fe_3_O_4_ composite nanofibers, respectively. Moreover, Energy-dispersive X-ray (EDX) spectroscopy results confirm the presence of oxide and iron within the fibers, as displayed in Fig. 2E, with an additional peak indicating the presence of carbon. The elemental composition of the samples is as follows: carbon (43.68%), nitrogen (45.83%), oxygen (10.35%), and iron (0.15%).

**Fig. 3:**
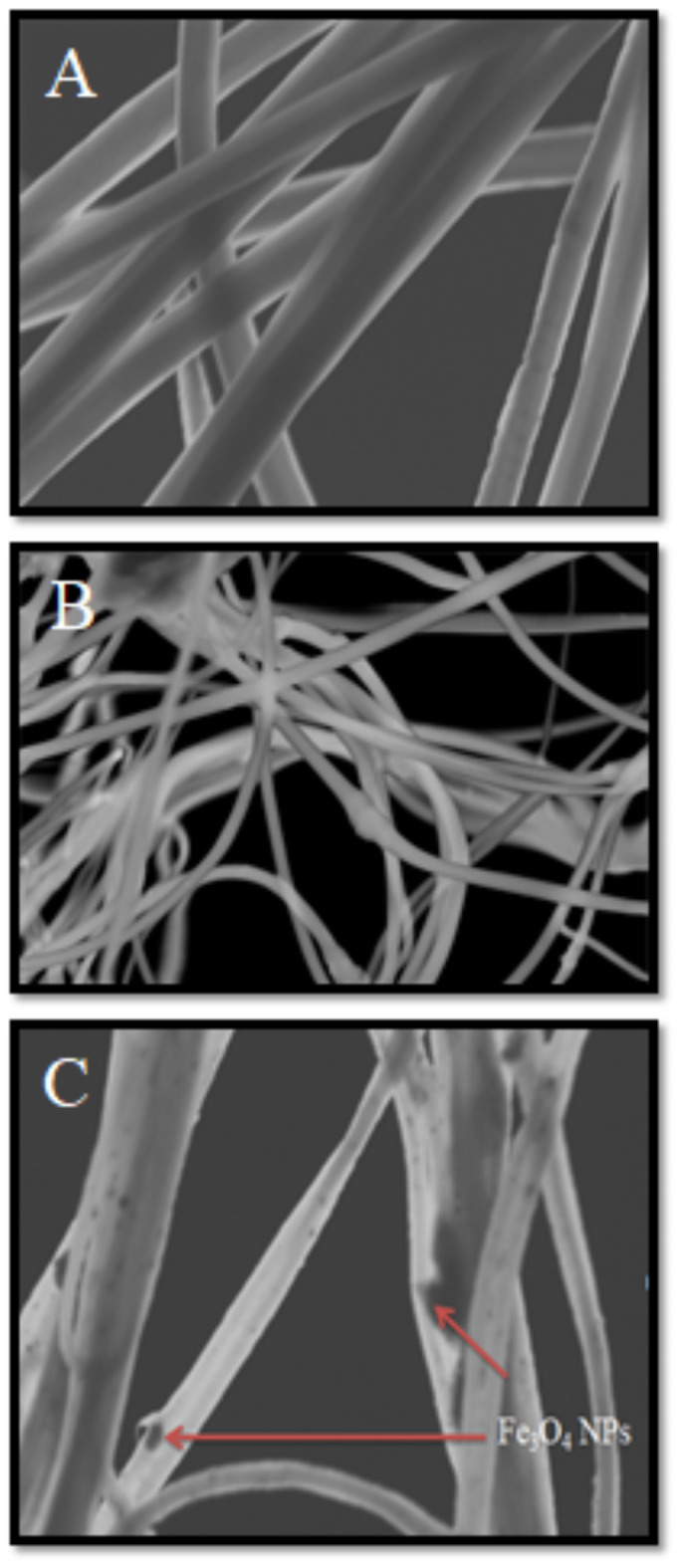
STEM micrographs of (**A**) PAN, (**B**) PAN-PPh. and (**C**) PAN-PPh./Fe_3_O_4_ nanofibers.

Additionally, STEM was employed to further elucidate the morphological characteristics of PAN, PAN-PPh., and PAN-PPh./Fe_3_O_4_ nanofibers, as illustrated in Fig. 3. Micrographs of PAN and PAN-PPh (Fig. 3A, B) indicate that the diameters of the nanofibers are not uniform. In contrast, the STEM image of PAN-PPh./Fe_3_O_4_ nanofibers (Fig. 3C) shows a more uniform structure composed of irregular aggregates of Fe_3_O_4_ nanoparticles, with an average particle size of 81.73 nm.

#### Thermal gravimetric analysis of nanofibers

**Fig. 4:**
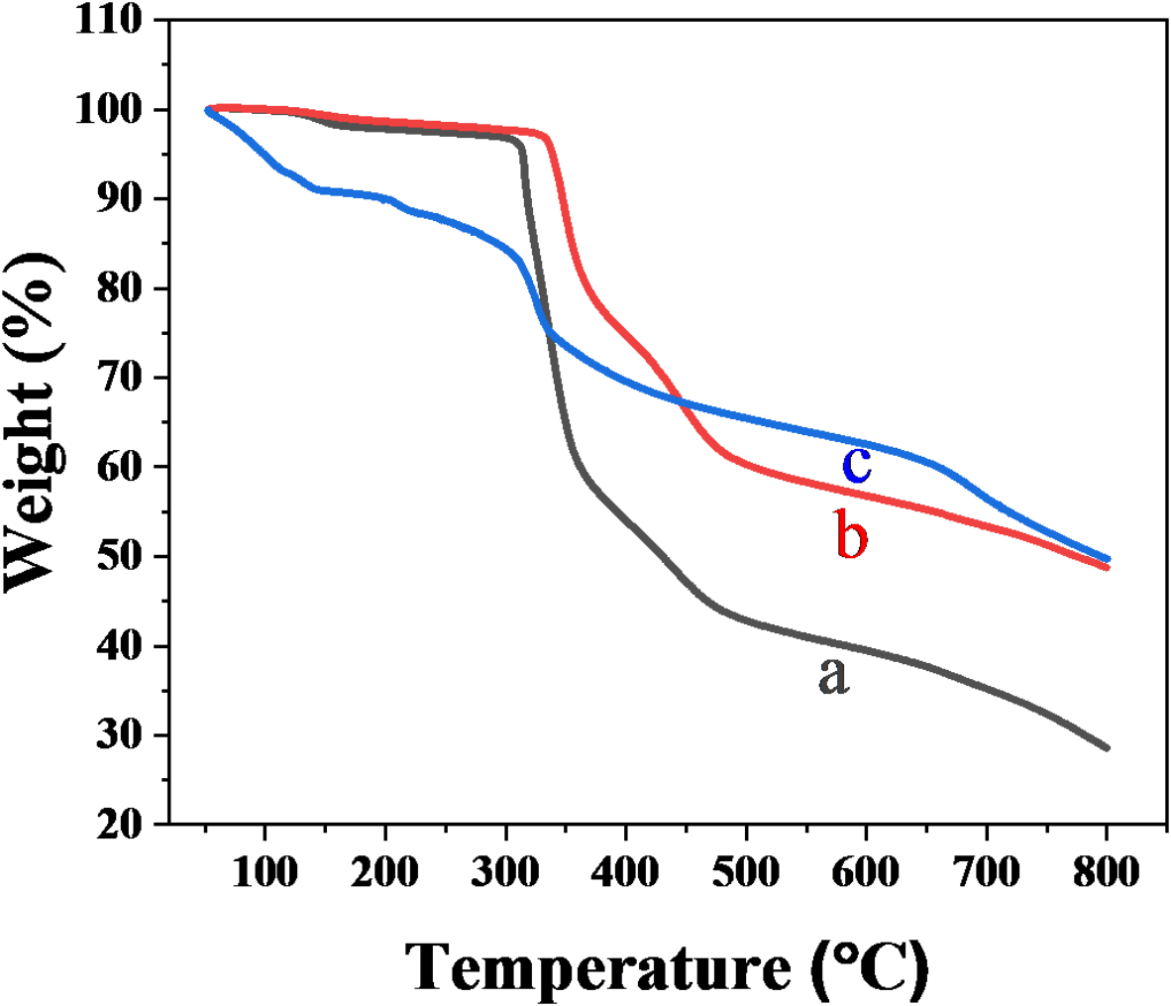
TGA curves of (**a**) PAN, (**b**) PAN-PPh. and (**c**) PAN-PPh./Fe_3_O_4_ nanofibers.

The TGA was conducted under nitrogen (N₂) atmosphere to assess the thermal stability of PAN, PPh., PAN-PPh., and PAN-PPh./Fe_3_O_4_ nanofibers, as illustrated in Fig. 4 and S_3_. The TGA results, displayed in Fig. 4, reveal that all nanofibers undergo thermal degradation between 50 and 800 °C, with total mass losses of 70.8%, 48.24%, and 49.29% for PAN, PAN-PPh., and PAN-PPh./Fe_3_O_4_ nanofibers, respectively. Notably, PPh. exhibits the lowest thermal stability among the nanofibers analyzed. The thermal decomposition of PAN-PPh. nanofibers is delayed compared to PAN nanofibers, attributed to the enhanced thermal stability associated with increased branching in the polymer and potential improvements from prior heat treatments in an inert atmosphere^40^. PAN-PPh./Fe_3_O_4_ nanofibers demonstrate moderate thermal stability, which is beneficial for the development of supercapacitors, likely due to the presence of Fe_3_O_4_ nanoparticles within the PAN and PPh. matrix^37^. In the initial and second stages of thermal decomposition, weight losses for the nanofibers are as follows: 3.44 and 36.74% for PAN, 2.5 and 18.58% for PAN-PPh., and 9.08 and 6.62% for PAN-PPh./Fe_3_O_4_. In the third and fourth stages of decomposition, weight losses are 16.43 and 14.49% for PAN, 16.38 and 10.78% for PAN-PPh., and 20.17 and 13.42% for PAN-PPh./Fe_3_O_4_.

### Electrochemical processing

**Fig. 5:**
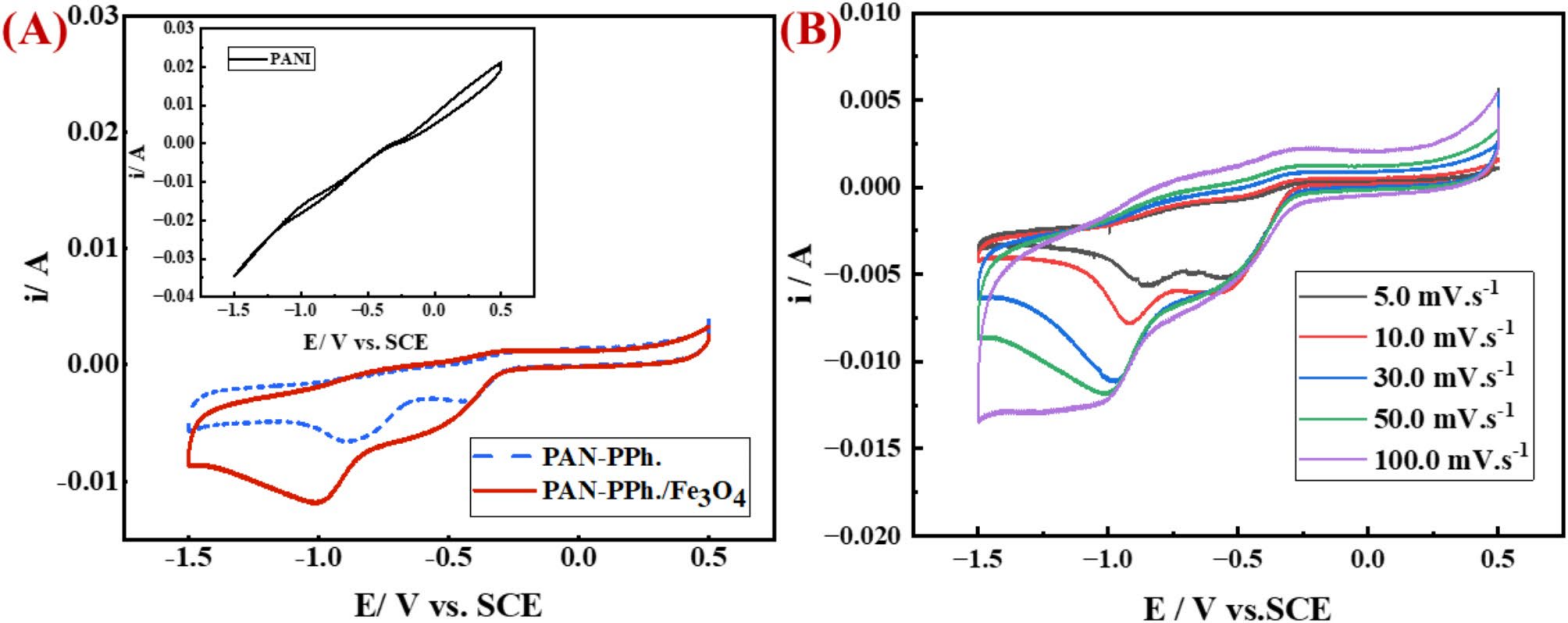
(**A**) cyclic voltammetry (CV) of (a) [PAN], (b) [PAN-PPh.], and (c) [PAN-PPh./Fe_3_O_4_] nanofibers GCEs *v* of 50 mV s^− 1^. (**B**) cyclic voltammetry (CV) of [PAN-PPh./Fe_3_O_4_] nanofiber electrode at various *v* mV s^− 1^.

The integration of novel materials into specific electrolytes has recently enhanced the electrochemical performance of supercapacitors. To evaluate the specific capacitance, resistance, and stability of the fabricated electrodes, several electrochemical techniques were employed, including cyclic voltammetry (CV), galvanostatic charge-discharge (GCD), and electrochemical impedance spectroscopy (EIS). Cyclic voltammetry (CV) was performed to investigate the supercapacitive behavior of [PAN], [PAN-PPh.], and [PAN-PPh./Fe_3_O_4_] nanofiber glassy carbon electrodes (GCEs), as shown in Fig. 5A. The CV measurements were recorded in a 10.0 ml solution of 1.0 M KOH, with a scan rate of 50 mV/s. The electrochemical process included Hg/Hg_2_Cl_2_ and Pt electrodes as the reference and counter electrodes, respectively.

The CV of [PAN] demonstrates a very low capacitance current and lacks redox peaks, as illustrated in Fig. 5A; inset. In contrast, the cyclic voltammograms of [PAN-PPh.] and [PAN-PPh./Fe_3_O_4_] nanofiber GCEs (Fig. 5A) exhibit enhanced electrochemical performance and notable capacitance characteristics, attributed to the conductive properties of [PPh.] and the presence of Fe_3_O_4_. As, the Polyphenyl components including [PAN-PPh.] may exhibit its own distinct electrochemical behavior. If the material contains conjugated structures or aromatic rings with redox-active sites (such as quinone groups or other electron-deficient centers), a reduction peak could emerge as the material undergoes a redox transition^41,42^. This process may involve electron transfer to the carbon framework or the aromatic rings and cause a reduction peak around − 0.5 V^43,44^, as displayed in Fig. 5A. Moreover, the reduction of reactive oxygen species (ROS) can also contribute to the observed peak due to the exposition of PAN-PPh. nanofibers to oxygen or have oxygen-containing groups on their surface (such as hydroxyl, carbonyl, or carboxyl), the reduction of these oxygen species could occur at a potential of approximately − 0.88 V^45^, as displayed in Fig. 5A. For example, the reduction of water to ROS has a standard reduction potential of approximately − 0.82 V, as described by the reaction:

$${{\text{H}}_{\text{2}}}{\text{O}} \to {\text{ROS}}\,+\,{\text{2}}{{\text{H}}^+}+{\text{2}}{{\text{e}}^ - }$$

Moreover, the doping of PPh. induces positive charge that is hypothesized to delocalize along the polymer backbone, creating a hole in the valence band and generating localized disruption defects. This results in a localized band gap between the highest occupied molecular orbital (HOMO) and the lowest unoccupied molecular orbital (LUMO) of the conductive polymers (CPs). The conjugated orbital overlaps in PPh. facilitates continuous electron transport along the polymer backbone, promoting efficient charge transfer when charge carriers are present^36^. Additionally, iron oxide is favored for its multiple oxidation states, extensive redox chemistry, and high specific capacitance^27^.

The CV curves of PAN nanofiber and magnetite (Fe_3_O_4_) exhibit properties corresponding to the pseudo-capacitance and double-layer capacitance of PAN-PPh./Fe_3_O_4_, respectively. In CV curve of PAN-PPh./Fe_3_O_4,_ the deviation from the ideal rectangular CV profile, characteristic of perfect capacitors, is evident with redox peaks appearing in both the negative and positive current regions. This deviation from optimal capacitive behavior is attributed to reduced contact between the electrode and the electrolyte^46^. Nonetheless, the persistence of redox peaks at high scan rates for PAN-PPh./Fe_3_O_4_ suggests enhanced charge transport through its network structure, as supported by morphological analysis^46^.

The specific capacity values for the [PAN], [PAN-PPh.], and [PAN-PPh./Fe_3_O_4_] nanofiber-based GCEs are 0.075, 0.147, and 0.246 Ah g^−1^, respectively. These values can then be converted into specific capacitance, yielding 135.5, 265, and 442.4 F g^−1^ for the [PAN], [PAN-PPh.], and [PAN-PPh./Fe_3_O_4_] nanofiber GCEs, respectively. Notably, the appearance of voltammetric peaks (P_II_, _III_) and (P_I_, _II_, _III_) in [PAN-PPh.] and [PAN-PPh./Fe_3_O_4_] nanofibers indicates an increased capacitance, primarily attributed to the aqueous electrochemical (Ox./Red.) reactions involving the phenolic groups of PPh^47^, and Fe_3_O_4_. The performance enhancement is influenced by the electrolyte energy levels and the overlap of electrical levels on the composite surface^48^. The [PAN-PPh./Fe_3_O_4_] nanofiber GCE demonstrates a significantly higher capacitive current density compared to [PAN] and [PAN-PPh.], indicating superior electrochemical capacitive performance.

Further analysis of the voltammetric response at various scan rates (5, 10, 30, 50, and 100 mV s^− 1^) is essential for evaluating the capacitive behavior of the proposed electrode. The CV of [PAN-PPh./Fe_3_O_4_] nanofiber GCE, as illustrated in Fig. 5B, reveals an increase in the current intensity of P_III_, accompanied by the significant decrease in the current intensity of P_II_ and a decrease in the current value of P_I_ at a scan rate of 50 mV s^− 1^. This observation demonstrates rapid electron transport on the electrode surface^49^.

**Fig. 6:**
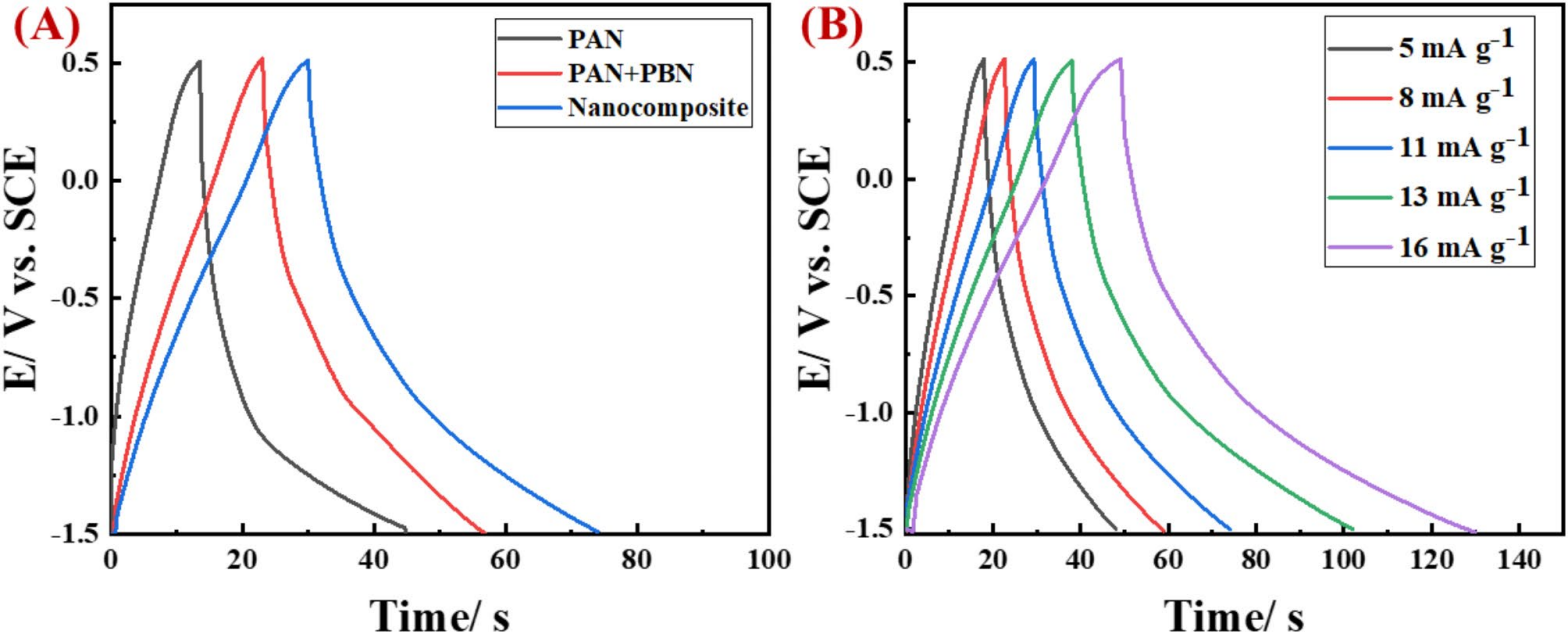
(**A**) GCD curves of (a) [PAN], (b) [PAN-PPh.], and (c) [PAN-PPh./Fe_3_O_4_] nanofibers GCEs, (**B**) [PAN-PPh./Fe_3_O_4_] nanofiber GCE at different current density A g ^− 1^.

As illustrated in Fig. 6A, the charge-discharge curves for [PAN], [PAN-PPh.], and [PAN-PPh./Fe_3_O_4_] nanofiber GCEs were measured in a 1.0 M KOH electrolyte at a current density of 11 A g ^− 1^. The specific capacity values measured for the [PAN], [PAN-PPh.], and [PAN-PPh./Fe3O4] nanofibers are 0.137, 0.173, and 0.229 Ah g^−1^, respectively. These values are subsequently converted into specific capacitance, resulting in 246.1, 312.23, and 412.5 F g^−1^ for the [PAN], [PAN-PPh.], and [PAN-PPh./Fe_3_O_4_] nanofiber-based GCEs, respectively, which are consistent with the CV results. Additionally, the specific capacitance of [PAN-PPh./Fe_3_O_4_] nanofiber GCE was recorded across a range of current densities A g ^− 1^, as displayed in Fig. 6B. The GCD curves demonstrate that [PAN-PPh./Fe_3_O_4_] nanofiber GCE exhibits specific capacitance values ranging from 264 to 704 F g^− 1^ at 5.0 to 16 A g ^− 1^, respectively.

**Fig. 7:**
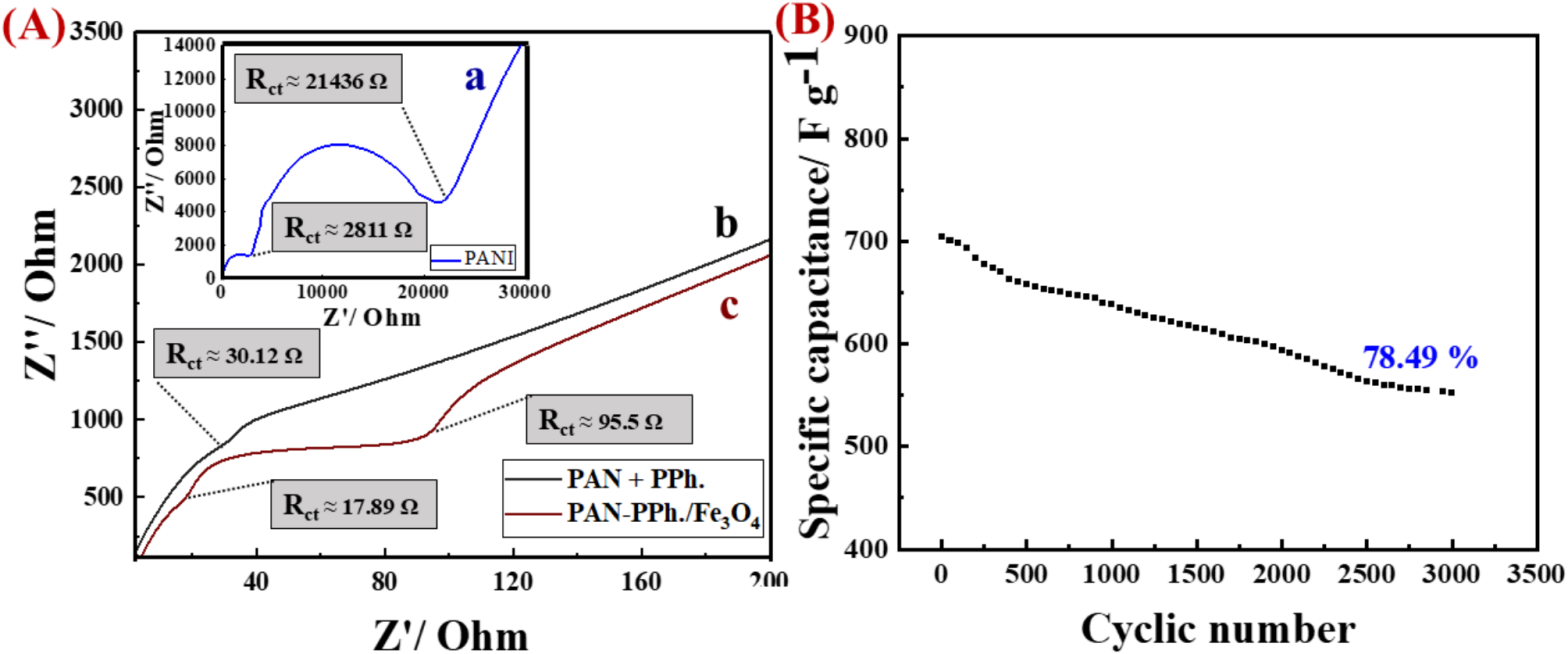
(**A**) Nyquist plots of (a) [PAN], )b) [PAN-PPh.], and (c) [PAN-PPh./Fe_3_O_4_] nanofiber GCEs. (**B**) Cyclic stability plot of the [PAN-PPh./Fe_3_O_4_] nanofiber GCE after 3000 cycles at a current density of 16.0 A g^–1^.

The capacitive efficiency of the proposed electrode is primarily influenced by the charge transfer resistance (R_ct_). To assess R_ct_, electrochemical impedance spectroscopy (EIS) and the corresponding Nyquist plots were recorded over a frequency range of 0.1 to 10^6^ Hz, as illustrated in Fig. 7A. The semicircles in the Nyquist plots represent the R_ct_, which reflects the surface characteristics of the electrodes^39^. The EIS measurements reveal R_ct_ values of 2811 Ω for [PAN], 30.12 Ω for [PAN-PPh.], and (17.89 Ω and 95.5 Ω) for [PAN-PPh./Fe_3_O_4_] nanofiber GCEs.

Furthermore, the stability of the [PAN-PPh./Fe_3_O_4_] nanofiber GCE was evaluated through a cycle life test for 3000 cycles at a scan rate of mV s^− 1,^ and a current density of 16.0 A g^–1^, as illustrated in Fig. 7B. The specific capacitance values recorded after the 1st cycle and after 3000 cycles were 1256.0 F g^− 1^, and 985.0 F g^− 1^, respectively, indicating a capacitance retention of 78.49% after 3000 cycles, as depicted in Fig. 7B. The low cyclic stability of the composite after 3000 cycles is primarily due to insufficient contact between the electrode and electrolyte, hindering charge transfer and deviating from expected capacitive behavior^46^. Fe_3_O_4_ enhances charge transfer through pseudo-capacitive behavior, but repeated cycles cause phase transformations, such as shifts to γ-Fe_2_O_3_ or α-Fe_2_O_3_, which have lower capacitance^50^. Mechanical stresses from redox processes can crack Fe_3_O_4_ nanoparticles (NPs), reducing the surface area for charge transfer and lowering capacitance retention. Additionally, the bond between Fe_3_O_4_ and the polymer matrix weakens over cycles, leading to detachment of nanoparticles and compromising structural integrity^51^. Environmental factors, such as temperature, humidity, and electrolyte, can further degrade interactions between Fe_3_O_4_ and the polymer, causing loss of cohesion and uneven dispersion, which reduces conductivity and capacitance. This disruption impedes charge transport and further decreases stability.

A comparative analysis of the PAN-PPh./Fe_3_O_4_ nanofiber electrode with other nanocomposites and Fe_3_O_4_-containing materials is presented in Table 1. The PAN-PPh./Fe_3_O_4_ nanofiber electrode exhibits strong cycling stability, maintaining 78.49% capacitance after 3000 cycles, making it suitable for applications requiring moderate stability. Although CNF-based composites like CNF/SnO_2_/PPy^52^ exhibit slightly higher capacitance retention (81.1% after 2000 cycles), the longer cycling duration of the PAN-PPh./Fe_3_O_4_ nanofiber electrode suggests better suitability for applications with longer lifespans. The Fe3O4@CNFMn flexible composite^53^ offers 85% capacitance retention after 2000 cycles and flexibility, ideal for wearable electronics, but has a lower specific capacitance of 306 F/g compared to the PAN-PPh./Fe_3_O_4_ nanofiber electrode. Compared to Fe_3_O_4_/PANI nanonets^11^, the PAN-PPh./Fe_3_O_4_ nanofiber electrode offers superior specific capacity (0.246 Ah g^-1^) and good electrochemical performance, though with slightly lower cycling stability. The Fe_3_O_4_/PANI nanonets^11^ achieve better cyclic stability (85% after 2000 cycles) but involve complex fabrication, making them less cost-effective for large-scale production. In contrast, the PAN-PPh./Fe_3_O_4_ nanofiber electrode is easier to fabricate, offering a good balance of performance and cost-efficiency, making it ideal for supercapacitor applications where affordability and long-term reliability are more critical than extreme energy storage capacity.

In terms of energy efficiency, the PAN-PPh./Fe_3_O_4_ nanofiber electrode outperforms the PANI/Fe_3_O_4_/carbon cloth electrode^16^ in both specific capacity (0.246 Ah g^-1^) and specific capacitance (442.4 F g^-1^at 1 A g^-1^), compared to (332 F g^-1^) related to the PANI/Fe_3_O_4_/carbon cloth electrode. While the PANI/Fe_3_O_4_/carbon cloth electrode offers superior cyclic stability, retaining 91% of its capacitance after 4000 cycles, higher than specific capacitance and capacity of the PAN-PPh./Fe_3_O_4_ nanofiber electrode, which make it more suitable for applications requiring high energy storage and moderate current densities. Moreover, the PAN-PPh./Fe_3_O_4_ nanofiber electrode also competes well against the NC@Fe_3_O_4_/CF electrode^54^, which excels in area and volume capacitance but has lower specific capacitance. This makes the PAN-PPh./Fe_3_O_4_ nanofiber electrode a more efficient choice for applications that prioritize energy storage per unit mass at standard current densities.

**Table 1:** Comparison of cyclic stability due to Fe_3_O_4_ and their composite.

Compounds	Cyclic stability %	References
CNF/SnO_2_/PPy//CNF/Fe_2_O_3_/PPy	81.1	^52^
CNF/SnO_2_//CNF/Fe_2_O_3_	70.3	^52^
Fe_3_O_4_@CNF_Mn_	85.0	^53^
PANI/Fe_3_O_4_ nanocomposite	71.9	^12^
Fe_3_O_4_/PANI nanonets	85.0	^11^
NC@Fe_3_O_4_/CF	88.5	^54^
Octahedral shaped Fe_3_O_4_ and α-Fe_2_O_3_ NPs	83.0	^55^
Polyaniline/Fe_3_O_4_ composite@carbon	91.0	^16^
PAN-PPh./Fe_3_O_4_ nanofiber	78.5	This study

Carbon nanofiber (CNF), tin oxide (SnO_2_), iron oxide (Fe_2_O_3_), polypyrrole (PPy), magnetic iron oxide (Fe_3_O_4_), flexible composite membranes with electrosprayed MnO_2_ particles uniformly anchored on Fe_3_O_4_ doped electrospun carbon nanofibers (Fe_3_O_4_@CNFMn), polyaniline (PANI), polyacrylonitrile-polyphenyl/ magnetic iron oxide (PAN-PPh./Fe_3_O_4_).

## Conclusion

In summary, a highly effective PAN-PPh./Fe_3_O_4_ composite nanofiber was synthesized by electrospinning PAN-PPh. with Fe_3_O_4_ nanoparticles. This composite was utilized to fabricate supercapacitor electrodes, exhibiting notable pseudocapacitance with a specific capacity and capacitance of 0.2458 Ah g^− 1^, and 442.4 F g^− 1^, respectively. The PAN-PPh./Fe_3_O_4_ composite nanofiber demonstrates an exceptional cycle life of 3000 cycles with a capacitance retention of 78.49%. These results highlight the enhanced electrochemical performance of PAN-PPh./Fe_3_O_4_, attributed to the synergistic effects of its components, the high stability of Fe_3_O_4_ nanoparticles, and the significant pseudocapacitance of PAN. Due to its straightforward synthesis, low precursor costs, and excellent electrochemical properties, PAN-PPh./Fe_3_O_4_ represents a cost-effective material for next-generation supercapacitors.

## Electronic supplementary material

Below is the link to the electronic supplementary material.

Supplementary Material 1

## Data Availability

The datasets used and/or analysed during the current study available from the corresponding author on reasonable request.
