# Supplementary material for: Electrospun polyacrylonitrile-polyphenyl/magnetite nanofiber electrode for enhanced capacitance of supercapacitor
Source: Sci Rep. 2025 Apr 28;15:14885. doi: 10.1038/s41598-025-97089-x (PMC12037743; doi:10.1038/s41598-025-97089-x)
Supplement: Supplementary file 1 — Supplementary Material 1 [file 41598_2025_97089_MOESM1_ESM.pdf]

Electrospun Polyacrylonitrile-Polyphenyl/Magnetite Nanofiber Electrode for Enhanced Capacitance of Supercapacitor

El-Refaie Kenawy[a], Youssef I. Moharram[b], Fatma S. Abouharga[b] and Mona Elfiky[b].

[a] Polymer Research Group, Department of Chemistry, Faculty of Science, Tanta University, Tanta, 31527 Egypt.

[b]Analytical and Electrochemistry Research UNIT, Department of Chemistry, Faculty of Science, Tanta University, Tanta, Egypt.

Corresponding author E-mail: *Elfiky_mona@science.tanta.edu.eg*

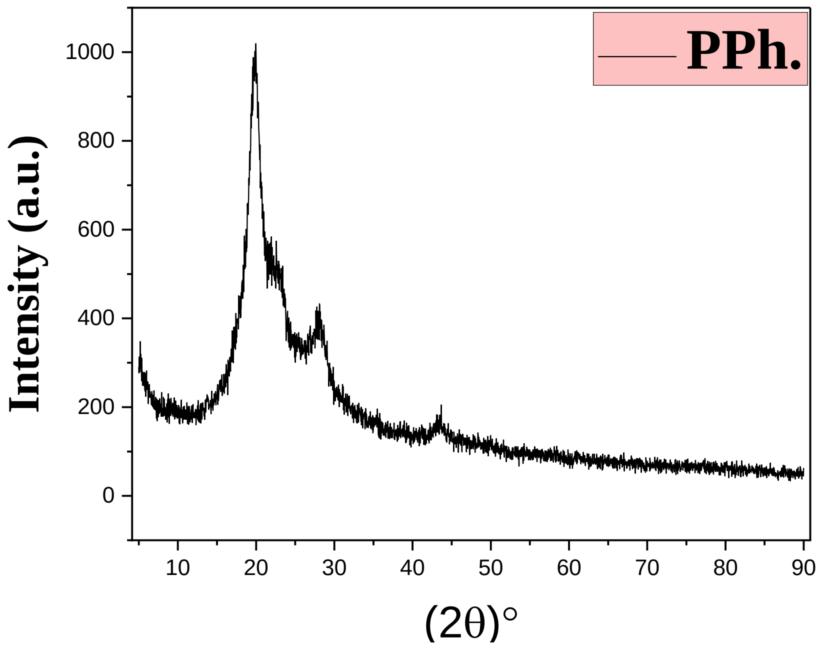

Figure S1. XRD pattern of PPh. nanofiber.

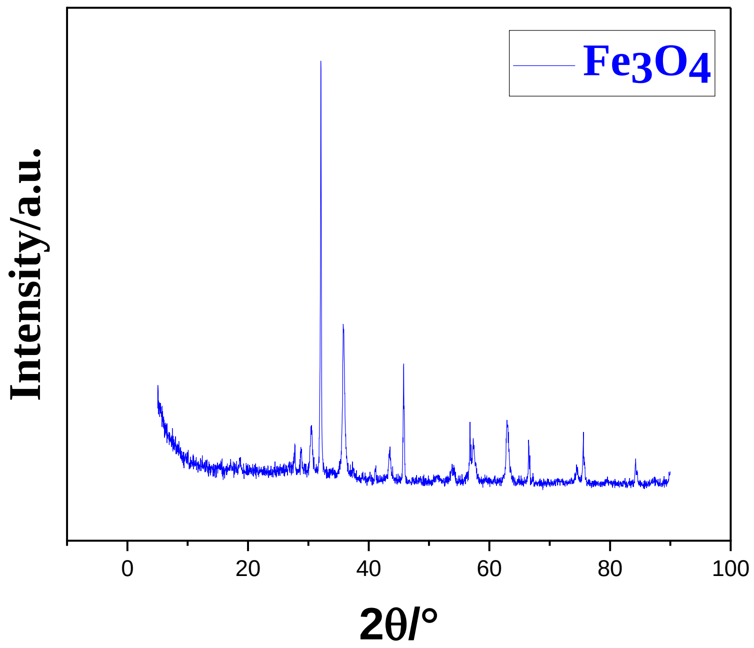

Figure S2. XRD pattern of Fe_3O_4 NPs.

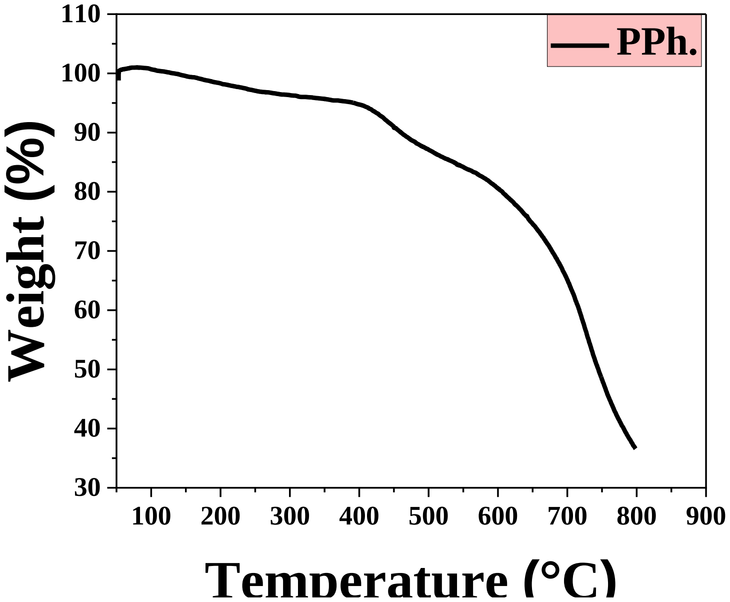

Figure S3. TGA curve of PPh. nanofiber.
